# The Necessity for Reform in Dental Education

**Published:** 1906-11-15

**Authors:** D. D. Smith

**Affiliations:** Philadelphia, Pa.


### THE NECESSITY FOR REFORM IN DENTAL EDUCATION.

BY D. D. SMITH, D.D.S., M.D., PHILADELPHIA, PA.

Read before the Michigan State Society, Detroit, July 12th, 1906.

It gives me pleasure to be present on this semi-centennial occasion, at the annual gathering of the representative intelligence of the dental profession in this great State of Michigan, and I am glad your meeting is in this beautiful city of Detroit, so widely recognized as the base of unimpeachable solidarities both in the field of medicine and dentistry.

I feel assured at the outstart of moral support from the dental section of your great University of Michigan, recognized as it is the world over, as the exponent of advanced dental education. I am sure that it will always second all efforts for genuine reform and better conditions for dentistry.

You will pardon me, if on this occasion I omit the stereotyped, adulatory exordium which is so commonly employed to set forth the achievments and wonderful advancement (?) of the dental profession in the past twenty-five or thirty years, and allow me, from my own standpoint, to present a view of dental conditions as I find them to-day. To secure this picture, I have pushed the dental camera a little closer to the object, re-adjusted some of the shade screens and brought the whole into a different, if not more perfect, light. My effort has been to focus attention more sharply upon some objects hitherto disregarded, perhaps unnoticed in the field of dentistry.

Possibly you will be dissappointed that I come to you with so little of compliment as to the present standing of the profession we represent, but I am not here in the spirit of the pessimist; on the contrary, I am an optimist, and believe that the dental profession will one day stand before the world as the coadjutor of medicine, a real adjunct to the mother science, and something more helpful to humanity than any present recognized branch of medicine.

Necessity for the existence of the dental profession is paramount; we occupy a field in human need unexplored and untouched by any other science. It is within the province of dentistry to contribute untold benefit to the comfort and well-being of humanity, and not to create or foster pathological conditions which perpetuate themselves, as inexcusable ignorance may allege.

These facts are without proper recognition solely because dentistry has failed to extend its confines and occupy its legitimate field of service.

Edward C. Kirk, D. D. S., Sc. D., of the Dental Department of the University of Pennsylvania, in an editorial of a comparatively recent issue of the *Dental Cosmos*, has taken the profession severely to task for its pereptual revolution around what he calls "The Same Old Thing."

He practically charges that the profession is made of up a lot of ignoramuses, deficient in orthography and good English, and who evolve "nothing new in dentistry." What new things can dentistry evolve, what new thing can it bring forth while its teachers dwell mainly upon and extol the filling of a hole in a tooth, the setting of a crown, or the construction of a dental bridge?

Dr. Horatio C. Wood, a most eminent professor in the same university, with great candor and the utmost boldness, has publicly declared the degree of D. D. S. to be "a badge of partial culture."

Professor Truman, editor of the late *International Dental Journal*, regaled us not long since with an editorial on the state of the profession in which he says: "We are floating in shallow waters; it would seem that we have not been doing all that is possible to meet changing conditions. It is quite evident that all this means for dental colleges a more stringent method, if not a new standard of training."

If the profession is revolving around "the same old thing," if it is "floating in shallow waters," if its degree is but "the badge of a partial culture," and this seems the concensus of opinion of the faculty of one of the leading institutions of the country, there must be something at fault somewhere. Is that fault discernable?

Dentistry is, or ought to be, the custodian of all the complex offices and conditions pertaining to the human mouth, and the human mouth is the very vestibule of human life; but dental teachers, dental writers and dental practitioners, seemingly of their own volition, have so circumscribed and limited the scope of dentistry, that it stands to-day for scarcely more, surely nothing higher, than the redemption of decayed teeth.

Have our schools of dentistry, or our representative practitioners impressed the medical profession or the public generally with the thought that dentistry represents anything beyond than the care of the teeth? Is it not true that in many cases, even extraction of these organs, the only operation approaching a surgical operation, as dentistry is now taught, is supervised by a medical attendant?

Let us here glance at what we stand for in the eyes of the outside world; Webster's Unabridged Dictionary defines "dentist" as follows: "Dentist, one who makes it his business to clean, extract and repair natural teeth, and to insert artificial ones."

The *Century*, that latest and supposedly most up-to-date standard in definition, defines "dentist" as "one whose profession it is to clean and extract teeth, repair them when diseased, and replace them when necessary by artificial ones, a dental surgeon."

Stripped of all embellishment and in cold type, these settings for the dentist and dentistry are not calculated to engender pride in our profession, or to make the dentist overly puffed up with the dignity of his calling.

Looking to the future, however, we may take comfort in the thought that in its true meaning dentistry will yet be a most important auxillairy to general medicine, and when it shall truly "come into its own" it will be esteemed, as we believe, as the most important branch of the healing art.

The service of dentistry will yet be recognized as a service to humanity greater than that of any present auxiliary of medicine. Why is it then, that we are persistently revolving around "the same old thing," that we are "floating in shallow water," that our degree is esteemed but "the badge of a partial culture?" What is wanting? Where does the fault lie? Is it not to a great extent in our methods of education?

No observer of present conditions will challenge the statement that there are already a sufficient number of colleges in this country granting the degree of dentistry; certainly there are quite enough to meet all present, or any immediately prospective requirements of the system of education at present in vogue.

The first dental college was instituted in Baltimore in the year 1839. The second, the Ohio Dental College, Cincinnati, was established in 1843. The third, the Pennsylvania College of Dental Surgery, came into existence in 1846. Since that time there have sprung up in various parts of the country some fifty to sixty similar institutions, and others are being added every year.

These institutions being equipped with an average of ten professors and minor teachers in each, are usually designated as "colleges." The word "college" varies in signification in different countries. In Scotland, the United States and in Canada, it is understood to mean, "an incorporated institution of learning of the highest grade.

If "college" it to be made to mean an institution of learning, even though the greatest latitude be granted the definitions, is it not strictly within bounds to say that few, possibly none, of our dental colleges are really entitled to the appelation? Can it be justly claimed that any of the fifty or more of our dental colleges have an equipment for teaching that should entitle them to recognition as institutions of learning?

Does the D.D.S., the D.M.D. or the Canadian L.D.S. under the present regime mark any degree of literary culture? Do any one of them denote any special attainment in any one of the sciences? Does any one of them carry with it any practical knowledge of either medicine or surgery? Can any one of them be said to be a badge of equipment for the practice of anything but the *mechanics* of dentistry?

Comparing the degree of dentistry with that of medicine, it will be found that they occupy widely divirgent standings in the community: they are widely divirgent in the attributes of authority and privilege. They are also regarded as far separated in educational culture.

There is not a question of diagnosis, treatment or general management, even in a purely dental case, where, if difference of opinion should arise, the dictum of the medical man would not be regarded as authoritative over that of the dentist. The M. D. is broad in the scope of its authority, and carries with it the right to practice in all fields. The D. D. S. confers no right of practice beyond the confines of simple dentistry. It does not permit of the signing of a death certificate, although death should result from causes which are strictly within the field of dental operations.

Who so bold as to deny that dentistry as represented by our schools and as practiced to-day, is not narrowed and circumscribed, even in its own recognized province?

Dental teachings in text books and periodical literature, instead of expanding the sphere of dentistry have norrowed it until it nowhere means much beyond the treatment of the one condition—caries of the teeth and its consequences.

Does not dentistry stand with folded hands and bated breath in the presence of the more serious troubles affecting the teeth themselves? Does it not acknowledge inability both in diagnosis and in the treatment of many baleful states of the human mouth, and conditions destructive of human teeth?

Foreign countries that a few years ago welcomed American dentists, are now practically barring out graduates who have only the dental degree from American institutions.

I am aware that it may be claimed that this is a matter of jealousy, or a selfish desire on the part of foreigners to stop American practitioners in foreign fields; but this explanation will scarcely satisfy observers who note the grade of scholarship and the advanced scientific work of some foreign institutions over American, and this, notwithstanding that they may note how inferior to Americans the majority of foreign operators are in their practical manipulations.

German endeavor is certainly leading American dentistry in experimental investigation and inquiry concerning the mouths of children in public schools. While compulsory inspection is by no means universal, efforts are being made in that direction, and plans for the betterment of mouth conditions are being considered. English medical diagnosis also, far in advance of American diagnosis, has discoverd untoward conditions of the mouth as causes of systemic disease.

It is along these lines that true oral prophylaxis will find its most perfect demonstration.

Since the unfolding of the great benefits of the oral prophylaxis treatment, perhaps a hundred patients have said in effect "your medical course helped you greatly, I suppose, in the development of this matter." On the contrary, in my medical course of two years (one year was credited to me for being a graduate in dentistry), I heard not one respectful allusion to dentistry, either as a special profession, or as a co-ordinate branch of medicine.

The study of medicine greatly enlarged the horizon of my vision. It brought more prominently into view the grandeur of the science and its great benefits to humanity. It shed new light upon its wonderful discoveries. It unfolded the self-sacrificing labor and toil of individual members of that profession for the good of mankind. It filled me with new aspirations and new desires. It also uncovered as never before the fact that medicine has its limitations. While it furnished no more extended insight into dentistry proper, it revealed the fact that medicine at its best estate is wholly ignorant of dental conditions and dental possibilities.

It taught me that medicine is as totally devoid of knowledge of dentistry as dentistry is without knowledge of medicine.

To the dentist becoming a student of medicine, the lack of homogeneity in teachings and practice is painfully apparent. The distinction appears as an impassable chasm; or one which can only be bridged by a better understanding and more consistent teachings on the part of both professions.

As an illustration of the want of apprehension of dentistry, or so-called dental surgery by medical men, let me mention that at a time now past one of the most distinguished surgeons of his day at one of his clinics, called me into the operaing arena to extract a partially imbedded right lower wisdom tooth, and presented me with a pair of straight superior incisor forceps with which to perform the operation.

So far as I have been able to observe, this is a fair illustration of the attentions given by surgeons and medical men to dentistry or mouth conditions.

But let me here present this prophesy; educated dentistry will yet demonstrate its power to aid medicine with an emphasis which shall gain for it not only recognition, but full respect and admiration.

There are innumerable claims and assertions to the effect that dentistry is at present a branch or department of medicine; such claims are wholly without substantiation; there is no practice in dentistry to show it.

"Father," said a thoughtful little boy, "how many feet has a dog if we call his tail his foot?" "Why, five feet my son." "No father, that isn't right." "Why isn't it right?" "Why, you see, calling his tail a foot doesn't make it a foot."

No more does affirming dentistry to be a branch of medicine, make it a branch of medicine. Unpalatable as it may be to our pride, the fact remains—dentistry as at present taught in our schools, is essentially a system of mechanics.

As a stream cannot rise above its fountain head, no more can a profession rise above the level of its educational institutions. The present methods of dentistry are distinctly an outgrowth of inadequate conceptions. Courses of dental instructions are not unlike the experience of a youth, wandering along a field footpath, who came upon a large stone inscribed on the upper side with the words "turn me over," and who, after much difficulty, finally succeeded in turning the stone over, only to find on the other side, "now turn me back again, so that I can fool some other idiot."

Phillips Brooks once said: "There are two elements in the making of every perfect work—a perfect pattern and a perfect workman." For very good reasons a perfect pattern for our profession has not yet come into view. The perfect teacher is perhaps too modest to announce himself.

There have not been in the past, nor are there in the present, adequate facilities for the preparation to teach the science of dentistry.

Of necessity, the important offices of instructors have to be recruited from the rank and file existing conditions; and the D. D. S. has furnished the larger quota. There are "professors" in our schools to-day flounting only the D. D. S., who are so puffed up with foolish conceit and self-importance, that they refuse to avail themselves of many opportunities for acquiring better methods and a broader culture.

Is not investigation looking to prevention better than being forever occupied in mechanical repair?

Listen to the song of Dr. A. G. Bennett, in the *Dental Digest*, July 1902, on

THE TRUE DEPARTURE.

"The merest mention of prevention
Brings out the true ideal,
And each resource and all our force
Combine to make it real.
Like the strenuous life with vigor rife,
With virile force abounding,
It has no need of any creed,
It does its own expounding."

To be successful in bringing students to an institution, it is assumed that the *mechanics* of dentistry must principally be taught; and thus it has come to pass that the "operator" the mechanic, frequently, with scant regard to merit or literary qualifications, has assumed the place of the teacher.

It was common report at the meeting of the National Dental Association at Asheville, N. C., in 1903, that a Dean, representing the dental department of one of our leading universities was censured and fined in the Faculties Association for violation of rules adopted for the matriculation of students. We may guess the reason why matriculates in dentistry very frequently do not measure up to even the low educational standard adopted by the Faculties Association.

It cannot be successfully disputed that the mechanics and mechanisms of dentistry are the dominating factors in the judging of the qualifications of a student or practitioner. If he can fill teeth, if he can "make a plate," if he can adapt artificial teeth in place of natural ones; in later time if he be adjudged a competent inlay, crown and bridge worker, deficiencies in scholarship in general science and in ethical culture are condoned or even entirely waived.

And must the service of dentistry forever remain the mechanical routine of crowding gold, amalgam or porcelain into cavities of decay? Must it ever be that the goal of our dental schools is to be the goal of numbers rather than that of education and usefulness?

Have dental departments in medical colleges and universities, from which, at their institution, so much of good was expected, improved the educational status of dentistry? Not markedly, if at all. If it should be urged that medical colleges have thereby at least *recognized* dentistry, with equal force, the same may be said of their attitude toward "Veterinary." The University of Pennsylvania in 1903, after joining with other colleges in a compact to advance the standard of dental education by lengthening the course of study, later made public announcement through its dean, Dr. Kirk, receding most ingloriously from the arrangements because forsooth "Harvard" refused to lengthen its course. It seems plain that dental departments—addenda to universities as at present maintained, are expected, first of all, to "draw" students.

If it was ever believed or expected that medical colleges or universities would make the department of dentistry the equal of the medical department in culture and training, that expectation has not been met. Even in the branches taught and studied in common, as chemistry, anatomy and surgery, there is good reason to believe that in most of these schools the dental student is neither required nor expected to be the equal of the student of medicine; and it would be difficult to conceive the dental professor as on an even plane in influence and in importance with the medical professor.

It is within my own and your remembrance that a medical faculty established a dental department and elected a dean who made his debut before the National Dental Association as the representative of his college, with a paper entitled "Dies and Counter-Dies!!" Can the true devotee of dentistry contemplate such conditions with much of satisfaction or hope?

Patrick Henry once said; "There is no way of judging the future but by the past." If this be true in educational, as in political matters, it must be admitted that there is not great encouragement to hope that in the near future there will be substantial elevation or gain in *matter* or *methods* in dental colleges.

With no intention or desire to detract from the importance of the practical application of mechanics in dentistry, we maintain that if the profession shall ever reach a higher plane and take its rightful place as a branch of medicine, there must be recognition of the vital necessity for a higher standard of scholarship and a broadening of the curriculum in every dental college and in every dental department in medical colleges.

The filling of cavities in teeth, whether with metals, cements or porcelain, must be subordinated to the study of conditions governing the *preservation* of teeth. The prevention of decay must be forced upon a level, higher than the mechanics of "extension for prevention." Overcoming and eradicating infection in the human mouth, must be adjudged more important and more to be desired than dental inlays, crowns or bridges. The *prevention* of diseased conditions must be esteemed greater than the cure of disease. The complex conditions and relations of the oral cavity must be studied and treated as the natural and rightful heritage of dentistry. Last, but not least, teachers must be prepared and competent to teach dentistry as a science, even as a department of medicine. Along these lines rather than in lengthening the general course of study, to educate, or in higher standards for admission, or in reaching after brighter students, we may confidently look for better things in dentistry.

There must also be reform amounting to revolution in the textual matter of dentistry. This is now so completely given up to the mechanics of dentistry, that neither the dental text book, our periodical literature, nor the didactic lecture offers much of benefit beyond the mechanics of filling teeth.

The present literature of dentistry exhibits nothing in common with the science of medicine or its teachings. Take the following as an example; In the latest text-book on operative dentistry, the first chapter, consisting of twenty-six pages, is devoted to a description of the physical characteristics of the teeth.

Two short paragraphs are given to, first, "definition," second, "function"; and the whole of the remainder of the twenty-six pages of the chapter to "mechanical design," of which the following is an extract:

"'The Lower Molars":—"The first lower molar approximates the second lower bicuspid on its distal side. It is the first of the two grinders of the lower jaw, and the largest tooth in the dental series. Unlike the upper molars the transverse diameters is less than the mesio-distal. The greater breadth is found across the base of the disto-buccal tubercle. The crown is square or trapezoidal in form, depending on the size of the fifth tubercle. Being quinquituberculate, the crown is broadened by the muilti-cuspid grinding face. The buccal face is inclined toward the center of the tooth for its morsal half to accomodate the occluding teeth. Architecturally, the tooth is formed of four cones and may be roughly divided into four quarters. There are four primitive cones with their tubercles and one cingule in the structure. The morsal surface (b) is trapezoidal in outline, the buccal line being the longest. The buccal angles are accute, while the lingual are rounded and obtuse. There are five tubercles, two on the lingual margin and three on the buccal. They are named the mesio-buccal, median buccal, disto-buccal, disto-lingual and mesio-lingual. These tubercles are less obtuse and more rounded than those of the other grinding teeth, mesio-buccal usually being the largest; the others are not so prominent, rarely raised and sharp. The ridges are: the marginal ridges, buccal, distal, lingual, and mesial, and the five triangular ridges descending from the five tubercles towards the center of the tooth." And so on and on for the balance of the twenty-six pages—not one whit more readible, instructive or important.

To gain a better idea of this text book, I may be permitted to quote from a critic of the work who dared to read it with impartial eyes; He says: "The mechanical make-up of this book is a credit to the publishers; the paper being good, the type large and plain and the illustrations well defined. That a number of the more important of the illustrations have been made to suit the fancy of the writer and are untrue to nature is not the fault of the publishers. In construction the work is after the plan of the "The American System of Dentistry," published by the same house in 1893, there being fifteen different writers for the twenty-three chapters or subjects treated. While something can be said in commendation of the work, it may be questioned whether collaboration in authorship is a special gain. The plan involves overlapping of subjects, repetition of matter and considerable diversity in methods of teaching, all of which is confusing to the beginner and of doubtful utility to the practitioner. These defects, however, are not so pronounced in this work as in the "American System."

"The authorship of 'The American Text-Book of Operative Dentistry" should warrant the statement that it embodies the latest and best in dental science, not only in discovery and practice, but in teaching also.

"As the claim that dentistry is a branch of medicine is sharply made in some quarters, we have examined this book with unusual interest, and great care, to determine whether this kinship, this link connecting the two professions could be discovered through this composite production of this body of dental writers.

"We have to confess that the claim seems wholly unsubstantiated in this, as it is in previous works upon dentistry. It is mechanics, mechanical manipulation and mechanical appliances that receive the consideration of every author, and are persistently at the front throughout this whole work. The opening subject of one of the principal writers is "The Operator," and the first sentence this: "The attitude of the body of the dental operator has considerable influence upon the ease with which the various positions required in operating may be assumed, and also has some bearing upon the freedom of the hands."

Omitting here much equally obvious detail, we come upon the highly entertaining sententce "the contact with the patient should be at as few points as possible, and should generally be made with the fingers." (Is this intended to advise the operator against the inadvertant use of the toes?) Another, a professor and dean, the man who so thoughtfully (?) warned the profession against oral prophylaxis lest the use of pulverized pumice and an orange-wood point, used once a month, should irreparably wear away the enamel of the teeth, begins his subject, "The Importance of the Proper Preparation of a Cavity for the Insertion of a Filling can scarcely be over estimated." We may, with great propriety ask ourselves here, is this the best that dentistry has to present to students of the science? If so, let us no longer boast of it as a *profession*, much less as a part of the medical profession.

"As if more fully to accentuate the mechanics of operative dentistry and perhaps to give prominence to special friends, instruments and appliances which have never found favor with dentists and are now obsolete, are illustrated and minutely described, while the great fields of etiology and prophylaxis are left practically untouched.

"In the chapter on 'Pyorrhea Alveolaris' following a succinct treatise on the history of this disorder, there is an apparent determination to circumscribe it within the bounds of certain papers which appeared in the *Cosmos* between 1892 and 1895, wherein this trouble was presented as a local manifestation of the gouty diathesis—constitutional.

"To support this theory important manifestations and facts have been overlooked or ignored, as have also some recent valuable contributions to the literature of the subject. Enough has long since been demonstrated respecting the etiology and treatment of alveolar pyorrhea to relegate such absurdities to the rubbish heap, instead of giving them place in dental text books to mislead students and retard investigation.

"Under 'Discolored Teeth and Their Treatment' the editor discourses upon discoloration in general with remarkable scientific acrobatism, but omits entirely to mention the most important phenomena relating to discoloration in tooth substance. As the medical man and the student of dentistry are alike interested in this subject, if at all, from a practical and helpful standpoint, the chapter on discoloration in this "American Text-Book" would have been far more complete and helpful if it had stated that owing to the greater vascularity of young teeth, they are far more liable to rapid and persistent discoloration than the teeth of adults, especially those of middle life.

"Teeth at this period exhibit greater density, with an apparent deposition of calcic matter and a consequent lessening of the vascular substance which forms the tubular and inter-tubular tissue. There are no discolorations of any moment from devitilization or other cause after the period of full consolidation. Satisfactory bleaching of a tooth, discolored from stasis or the fixation of decomposable matter in the tubular and inter-tubular substance, is an impossibility; therefore, he, who by good management, prevents discoloration, is doing a service far greater than he who attempts an impossible bleaching.

"The chapters on extraction of teeth are well written and satisfactory. In the extraction of teeth and only here medicine and dentistry seem to come to a common focus. From this point begins their divergence."

But it is not to the errors and antiquated teachings of our text-books only that we appeal to justify the position that reform is essential and demanded in the teachings of dentistry.

Turn with me to some of the misconceptions and errors that are ingrained in dental thought and teachings, some that have been so incorporated into its substance that they have become part of its warp and woof.

The first of these relates to the office of the teeth in our modern civilization.

In defining the offices of teeth, emphasis is universally placed upon mastication as the chief function of these organs. I am aware that criticism will be aroused when I say that this teaching should be abandoned and something more in consonance with real conditions substituted.

While the teeth are organs of mastication and away back in the savage state where practically a necessity to this function. In our present civilization they no longer stand in this relation.

Digestion, assimilation, maintenance of human life and health are attainable in a degree of perfection without tooth mastication. Indeed, there are many cases of infectious mouth conditions where conservation of health would be more easily and better maintained without teeth than with them.

The ordinary mouth with natural teeth carries a tooth surface that may be roughly estimated at twenty to twenty-four square inches in extent, varying with the size and number of the teeth. This large extent of tooth surface from lack of care in whole or in part, is very generally in a state of virulent infection.

As this surface is exposed to much of the inspired air and washed and mopped in mastication by direct contact of foods, it will be readily seen that the amount of infection carried into the pulmonary and digestive tracts will depend largely upon the state of the teeth and their surroundings, whether septic or aseptic.

Perhaps a more vivid picture of this may be presented by considering for a moment the condition of an unwashed plate, spoon, knife and fork. Such an outfit for serving food presents scarcely more square inches of surface than an ordinary set of natural teeth.

Imagine conditions which compel or admit of serving food from a plate covered with infected mouth fluids, decomposed food remains, viscid, mucoid secretions and excretions, occasional drops of pus, disengaged particles of calcic deposits, the whole impregnated with odors from tooth decay, putrescent pulp tissue, bacterially infected tooth surface, and other septic conditions represented in the unsanitarized human mouth, and we have but another view of states and conditions presented in the oral cavity, the vestibule of life; too often, through ignorant inattention and neglect, converted into a veritable vestibule of death.

Day by day and hour by hour septic matter is swept into the digestive tract to re-appear in days or months, or years, perhaps, in some form of systemic infection, and all because of the presence of natural teeth in the mouth.

Does it not thus become evident that in many cases mouth conditions are a bar to the conservation of health, and that, unrelieved of septic conditions, the system may be better without teeth than with them.

Full preparation of digestible, assimillable, nourishing foods is readily obtainable without aid from the teeth. However convenient and pleasurable the mastication of food may be, the preparation of food by the teeth is by no means a necessity. This is fully attested in the case of invalids so often nourished and brought back to health with pre-digested foods or foods prepared for the stomach outside of the mouth. It is also equally well attested by thousands upon thousands of the fully edentulous mouths.

If we seek out the old people of to-day we shall generally discover them with few or no natural teeth; and it is a matter of general occurrence that they have come into a better state of health as a result of parting with all of their natural teeth.

Where then, is the emphasis to be placed on the uses of the teeth? When dentistry shall fully apprehend the facts, the emphasis will not be upon the necessity for teeth in mastication, but upon the pleasure they afford as an adjunct to mouth mastication.

The pleasures and delights of the table have been celebrated in all civilazations. Climes, purses and service have been laid under tribute to please and gratify the taste. No act of friendship is more highly regarded than the hospitality of the table. Wherein do the teeth contribute to these delights? Largely by contributing to the pleasures of taste. The comfortable, unconscious use of the teeth in mastication affords a stimulus to gustatory pleasure and contributes a nervous aid to digestion which, in importance to the system, far exceed any demand for the exercise of the merely mechanical function of mastication.

Our literature should also be freed from the error expressed in the thought that caries of the teeth is a *disease* and from such pedantic and meaningless deliverances as that which sums up an article of eleven pages in the *Dental*

*Summary*, which says: "The predisposing factor in dental caries is diathetic and local treatment alone is inadequate to arrest it."

After more than fifty years of fairly successful effort in arresting decay by local means alone, this declaration must come as a distinct shock to the dental profession. It puts a bar squarely across all our past efforts and stamps every known method of treating decay as inadequate. The condemnation, as it seems to me, is not in failure to arrest decay by local means but in failure to do anything looking to the prevention of decay. If local treatment alone is inadequate to arrest decay, what is its twin help-meet? Why are we not assisted by our author to discover it?

Dr. Talbot complains that dentists are not readers. In view of much that is written, the question may be fairly propounded, are they thereby great losers?

Co-existent with the above and even more damaging and belittling to the profession is that all prevalent impression that decay is the principal and practically the only pathological condition with which dentistry has to do.

Teachers see nothing and apparently care for nothing beyond this. In consequence the life of the student and the practice of the practitioner centralize around the treatment of decay in the teeth. Decay no more belongs to the teeth than to other osseous structures, save as the teeth are independent bodies and in the half open, half closed cavity of the mouth, are subject to constant varying environmental conditions.

Truth demands the uncovering of the fact that the decay of the teeth is not a disease; it is but the result of chemical activities and affinities which, in favoring environment, are working upon the dissoluble osseous organs in the oral cavity. In our search for the discovery of some occult cause for decay we have failed to recognize the fact that chemical activities are governed by precisely the same law of intensity and arrest that control these activities and affinities in other fields.

Pernicious tooth environment attended always by lactic or other acids and heat intensifies the destructive affinities to which the teeth are subject, and thus the condition we call decay is established and perpetuated.

Environmental states alone may cause rapid disintegration and decay of the teeth, or environmental states for the same teeth may lift the whole into complete immunity. In the true sense, the forces operating as the cause of caries of the teeth have not a dual relation.

A more recent, but a more pronounced error which has gained notoriety through our periodical literature, is the attempt to make saliva a factor in, if not the prime cause of tooth decay, as well as to make it a purveyor of disease in the human mouth.

While mouth fluids, especially nocturnal mucus, are often exceedingly destructive to tooth substance, *saliva* in its action on all tissues of the mouth, not excepting the teeth, is most benign. There is no ingredient or element in it harmful or destructive to these organs. Little attention has been given by either medicine or dentistry to the mouth fluids generally known as saliva. Medicine has analized what it calls saliva, and ascribed to it the initiatory function in digestion of converting starch into sugar.

Saliva, the secretion from the salivary glands, really has just two offices, first, that of moistening and liquifying the food, while it is in process of mastication; and second, that of enveloping the bolus in a smooth, mucillaginous coating to facilitate deglutation. Saliva is often spoken of as a fluid constantly present in the mouth. On the contrary saliva appears at stated intervals only and then only during the taking and masticating of food. Saliva comes from three separate sets of dual glands, so respectively situated as to conveniently discharge their separate secretions into the mouth. The secretion from these glands is by no means a homogeneous fluid.

The largest of the salivary glands is known as the parotid; these glands are situated one upon each side of the face just in front of the ear; these glands have a crow-quill like duct, opening into the mouth in the buccal mucus membrane opposite the first superior molar. They discharge their secretions into the mouth in considerable quantities (often in a stream) to satisfy the demands of mastication. It is a fluid limpid as the clearest water.

The second set of glands, the sub-lingual, open into the mouth under the tongue just back of the lower incisors. The secretion from these glands is also limpid and apparently identical in character with the parotid secretion. The office of the fluid from these four glands is purely that of moistening the food in mastication and for its solution.

The third set of g'ands, known as the sub-maxillary, are situated under the tongue opposite the first and second lower molars. These glands discharge their secretion into the mouth through ducts known as the ducts of Wharton. The office and character of the submaxillary secretion is wholly different from that of the parotid or of the sublingual. This fluid is never poured out to be mingled with the food in mastication. It plays no part in mastication proper. It is the saliva of deglutition and is never discharged into the mouth except in the act of swallowing. In character it is a thick, distinctly ropy fluid, and when isolated as is frequently done in operations upon the lower first molar teeth, its mucillagious or ropy character is distinctly recognized. Not infrequently it may be seen extending from the mouth of the patient even to the floor and having the appearance of a rope of mucillage. It is this normal state of the submaxillary secretion that has given the impression that certain mouths produce a ropy saliva very prejudicial to the teeth. Nothing could be further from the truth. This secretion is ropy in its normal state, and is discharged intermitantly in considerable masses just as the bolus is leaving the mouth in its passage into the pharynx and esophagus. The raising of the tongue in the act of swallowing ejects a mass of this secretion which acts to envelope and lubricate the bolus in its passage to the stomach.

In normal conditions this secretion never reaches the anterior portion of the mouth; it does not even mingle with the food until in the act of swallowing. Its office is distinctively to assist and facilitate deglutition. We believe further investigation will show the secretion from the submaxillary glands to be the only part of the saliva having perceptable influence in the digestive process. If starch is converted into sugar by saliva it can be due to nothing but the chemical action of this odoriferous and highly impregnated fluid from the submaxillary gland. The parotid and sublingual secretions can have no more immediate influence than so much water.

A fact to be noted in connection with the salivary glands is the promptness and rapidity with which the secretion is taken from the circulation. They have no store house or reservoir; they secret only to the extent required and yet the supply for the demands of mastication is ever ready. The limpid salivary fluids might,without disadvantage, give place to water, or other customary drinks in many states of the mouth.

Water is not simply a food adjuvent, it is a diluent to the circulation and a necessity to digestion. It is an integral part of nutrition as necessary to digestion as it is to tissue replacement or the maintenance of life.

With cessation of mastication there is cessation of secretion on the part of all salivary glands. This function is resumed only upon natural restimulation through presence of food, or by some unusual interference.

Operations upon the teeth frequently excite profuse flow from some one or possibly all of these glands. It is not uncommon for the parotid and sublingual to pour out their limpid secretion in large quantities, while the submaxillary is wholly undisturbed. Again operations upon the lower jaw in the region of the submaxillary glands, especially upon the first and second lower molars, will cause it to pour out a continuous ropy stream, while the parotid and sublingual are seemingly dormant.

It will thus be seen that the term "saliva" applied to the secretion from the so-called salivary glands is a very uncertain designation. The scientist who speaks of "analizing saliva," while failing to distinguish the gland from which his specimens are taken, shows himself to be dealing most unsatisfactorily and unscientifically with the conditions that confront us. The limpid fluid from the parotid and sublingual glands can have no more influence upon decay in the teeth than so much pure water. The secretion from the submaxillary gland is of such a character and thrown into the mouth at such intervals, that it never comes in contact with the teeth; we can, therefore absolutely eliminate *saliva* in all its forms as a factor in the production of decay in the human teeth.

Saliva is a wholly different substance from that scanty viscid fluid constantly present in the mouth. These mouth fluids are principally a product of the mucus folicles—small glands situated in large numbers just beneath the mucus membrane especially of the upper lip. This folicular secretion, jointly with certain excretory fluids which give natural moisture to the lips and mouth is constantly present. But it is wholly different in character from the salivary secretion we have been considering.

Saliva in health is alkaline, mucus is acid. It is principally this mucus ever present when the mouth is at rest that has been so much analized of late by unfledged scientists, as saliva. Periodicity in the flow of saliva, permits practically continuous activity of the mucus secretion and thus, undisturbed, *it* becomes a prime factor in tooth decay, and not less in other dental troubles. Its normal viscidity furnishes the most favorable conditions for retention of acidulated matter in contact with the teeth, in situations where decay begins. Solution of enamel and dentin, which we call decay, is a result of the perpetual presence of these viscid mouth fluids in contact with the teeth. Tooth substance will yield more or less readily to the power of these solvents according as the teeth are vascular or compact in structure. It may, therefore, be justly claimed that decay of the teeth is wholly the result of environmental conditions. Neither medicine nor dentistry can, by any possibility, reach the conditions upon which decay is dependent by other than "local treatment."

We have not, at least now, the time to consider the many crudities and absurdities of lesser import that clog the wheels of progress in dentistry. Enough of a practical nature as we hope has been presented to demonstrate the proposition which forms the subject of this paper. Practitioners however need not await the advent of these reforms; they will appear in due course, both in our schools and in our literature.

One reform of greatest moment to ourselves and our patients stands ready for adoption at the hands of all who will. *Change of environment* for all teeth and all mouth conditions is the one effective weapon with which to combat the inroads of decay and meet the crying needs of a suffering humanity. Humanity is suffering physically, mentally, morally through long years of bondage to an unrecognized toxic mouth infection—preventable, but unrelieved.

Change of environment, frequent, forcible, thorough, is the watchword for relief, which should be rung out through all the confines of dentistry.

For "extension for prevention" substitute in practice "*extention of prevention,*" and thus stand face to face with the oral prophylaxis treatment in all its masterful significance and all its relief giving benefits.

### DISCUSSION.

DR. ZEDERBAUM, of Charlotte: I do not believe that physicians should tell us what we should or should not do. A patient will come to us and say, Dr. Blank said I should not take gas, or that I had better take chloroform. I believe it is up to us to decide whether we should give any or no anesthetic. Many times after you have completed some extracting operation patients will go for further treatment to the physician who hardly ever knows anything about how to treat such wounds.

Teaching in the dental schools from start to finish is not what it ought to be. I notice that several new subjects have been added to the curriculum recently. The course ought to have been extended to four years and the requirement for admission ought to be more severe. The question arises, what shall be done with the men who are naturally good mechanics from every standpoint and can render good service quickly, but are deficient in professional culture? I know many graduates who cannot explain which was first formed, the enamel or dentine; still, they will do a better technical piece of work than those who do know.

The teachers in our colleges should be of higher standard. In European countries they have better teachers. They are more thorough in whatever branch they take up along scientific lines.

Dr. Smith came here for the purpose of leading up in h's article to the point of prophylaxis. He will find there is a prophylactic squad already in Detroit. I would like to ask Dr. Smith, why is the mouth with the saliva (or decloudation?) as he terms it, of so unpleasant a smell; and, also, if it is a fact that the saliva in the mouth is present only during the time of mastication. Why is it presented in abundance when we have the heartburn. There are many more questions which could be placed before Dr. Smith, which I am sure could be answered positively.

Dr. Hoff, Ann Arbor: I suppose it is up to me to defend the college and the teacher. I presume I was put on for this purpose. Are the dental colleges teaching up to the profession's attainment? The inference is that we are not, as the essayist says, that the stream cannot rise higher than its source. Why should you expect your colleges to teach something that the profession does not intend to practice? Or why should you expect your dental colleges and teachers to know things that the profession does not know, and if they did know them, how are they going to get these things into the minds of your students, who do not know how to discriminate any better than you do? We cannot teach what the profession does not know, practice or endorse. I believe the colleges are teaching as nearly as is practicable for them to do up-to-date. It is not practicable, under existing circumstances, for the dental colleges to do very much better teaching than they are now doing. You know the reasons just as well as I do. Our colleges are not endowed; we have no training schools for training teachers of dentistry, such as we have for training teachers in theology. The colleges ought not to be blamed for not doing something that it is impracticable for them to do. The dental college has done a grand work. If it were not for the dental college with its history of the past fifty years, I do not think our profession could occupy the position it does to-day. I do not believe, in the remarks that Dr. Smith has made on this point, that he intended to discredit our profession in the sense that seemingly he has done. We know that he has honored it by giving himself to it, and I have no doubt he is proud that he is a member of it. I do not believe that if he had again to make his choice to-day, that he would take up the practice of medicine in preference to the practice of dentistry. Personally, I have never been tempted to take up the practice of medicine, go into the pulpit or wish I could go into the banking business. I am satisfied with the field of dentistry; it is big enough for me. What little I know about dentistry I hope to make useful to the people who call upon me for it and need it. I believe that in practicing the profession of dentistry, I can render just as good service in kind and just as humanitarian and divine a service as any clergymen in the city of Detroit, so far as my resources enable me. Dentistry is not a menial calling only so far as we make it so by our incapacity or indifference, and every conscientious teacher of dentistry is doing the best he can by precept and practice to instill this doctrine

The essayist tells us that college training is too largely given up to the development of the technical or mechanical department. I do not think that is true. I am using what knowledge and strength I have as a teacher to develop the technical department, and I feel that we cannot develop it too rapidly or too highly. There is so much of the technical involved in the practice of the profession of dentistry that we cannot ignore it. If we do, we shall lose our power and influence for good. Dr. Smith uses the technical methods in all of his work. What would his work be without the technical knowledge and training that he has? A gentleman who knows him, perhaps better than any one else, said to me that he was the best all-around dentist that he had ever known. I should like to have some one say that of me. I do not mean that we should not also want to be dental scientists; but to be an all-around dentist, able to render dental service of any character and of the highest possible ability, ought to make any man feel that he was indeed filling an important place in the world's work.

I say that we cannot ignore the technical or mechanical of our profession, if we do, we are lost. There is so much involved in the technical part of our profession that it is impracticable for us to ignore it, Ours is a technical profession, an artistic profession, and it can also support strong claims to being one of the scientific professions. It involves more knowledge, it seems to me, of the general sciences than many other professions. Surgery is a technical profession in all its branches. What would a surgeon be without his mechanical ability and skill? The surgeon does not depreciate the mechanical aspects of his profession. The man who is the best technical surgeon is the man who is most successful, providing he also has a good knowledge as a basis for his practice.

Dr. Smith has made a criticism of our text books and literature. I think it deserves a good deal of criticism. I know a good deal about text books and literature. I have been studying them ever since I have been in the profession. There is, perhaps, no text book on any subject in all our curriculum that is satisfactory to any teacher. Even the teachers who write the text books themselves are not satisfied with their own books. They cannot be because our knowledge and technique is growing so rapidly and making such advances in all directions, that no text book three years old can be of any considerable value. This may appear to be a singular statement for me to make, but it is a fact that the text books cannot possibly keep up with the professional progress that is being made. The text book on crown and bridge work that is five years old is almost out of date. Of all works on practical dentistry the same may be said.

We have made all kinds of advances within the last fifteen years; I veritably believe, that no profession has made such advance in the same amount of time as has dentistry. I do not feel that we ought to depreciate ourselves or our profession in any way, or feel that we are not making the progress that we should make.

Does the dental degreed student have only partial culture? Of course he has not. You could not make me believe that if you said it all night. I am surprised that Dr. Smith would quote the statement of such a man as made the above statement. Dr. Woód does not know anything at all about dentistry, the inner life of it, or what it means to be a dentist, or he never would have made such a statement. In a sense, it is partial culture just as much as medicine, theology or law, or any other profession has only partial culture. Any professional study must be in a sense only partial culture. because we cannot have broad and general culture except we spend a large portion of our lives in the pursuit of academic studies in the sciences and arts. How many men have found the time or inclination to do such a thing, to say nothing of the capacity, and if we had all general culture, of what value could we be in the world?

We would be of no more value than the man who has piled up millions of dollars and never uses a cent of it for the good of man-kind. A little knowledge of many things does not stand for culture, but rather a comprehensive knowledge of a few things will give one a reputation for wisdom and culture.

There are dentists who have only partial culture, just as there are medical men and preachers and other men, who are only partially cultured; but dentistry is capable of furnishing a man with the broadest kind of culture. There is opportunity in any department of dentistry for a man to become broad and cultured. If we consider the subject of orthodontia, or any other of the specialties of dentistry, even prophylaxis itself; who is there here to-night who has heard this brilliant lecture of Dr. Smith, that would feel able in any limited amount of time to compass the study of prophylaxis and its treatment that he has pictured to us, and yet it is only one of the aspects of dentistry. Dr. Watson will tell you what a broad field orthodontia opens up for us. Dr. Oakman has made heroic sacrifices during the last three years, in more ways than I can tell, to get a better knowledge of general medicine that he may practice oral surgery. If you will ask him what he has gone through, what sacrifices of time and money and energy to get this knowledge, you will get some idea of what it means to be a cultured man. When you expand that out into the broad field, who is there of us that is able to do it.

Does the dental degree give its holder the right to practice medicine? Of course not; but who wants to practice medicine? The practice of medicine is an art of itself. It may be true that the general public goes to the practitioner of medicine and gives him a higher appreciation for his opinion on diagnostic subjects for instance. They even say for these medical men that they have a right, perhaps, as Dr. Zederbaum has said, to advise the dentist what he should do. I have no objection to a practitoiner of medicine advising me, I am very glad to have him do so. I very frequently call upon my medical friends for advice, because I know they are in a position to diagnose conditions that I am not. I am very glad to consult my medical associates, just as I am to have my dictionary to consult for words I do not know how to spell. There is no need for the dentist to take on the practice of medicine. We have all we can do well in our own field. and we are more capable of practicing dental medicine than any medical practitioner can be, because we are dealing with dental diseases and the application of remedies for diseases that the medical practitioner knows nothing about. There is not a man in this presence who has practiced dentistry for any time who has not seen cases of mal-practice in dental treatment coming out of the hands of medical men. I know of two eminent medical practitioners who have made decidedly wrong diagnoses of two dental cases and failed in their treatments for lack of special training. When these cases were finally referred to a dentist, they yielded promptly to a proper treatment. You will find that men who are specializing in medicine do not desire to be advised by another medical man who is specializing in some other field, but, at the same time, he he is very willing to take advice when he does want it. As far as the dental degree giving the holder the right to practice general medicine, I think it would be a great hindrance to both medicine and dentistry, no man can do both as well as the people have a right to expect. We ought, however, to practice dental medicine, and practice it scientifically as well as skilfully.

Dr. Smith makes the statement that true dentistry is yet to become the most important branch of the healing art. This is rather a strong statement, but he refers, of course, to his specialty of prophylaxis. I believe that the statement may be well within the bounds of reasonable prognosis in regard to the specialty of prophylaxis. It may be true that prophylaxis is going to help us to become a recognized specialty of medicine whether the medical practitioners want us to be or not. We are going to be so important a factor in the treatment of diseases of the human body that they cannot help recognizing us. But our recognition is not coming alone from prophylaxis. The whole range of the practice of dentistry will have an important bearing. We can look forward to this prophylaxis treatment along with other dental accomplishments as great and powerful levers which are to help us to that recognition. It probably is also true that we are not making such progress as we ought to make; but we have made considerable progress for the time we have been at the business.

Dr. Smith says that no work has been done so far in the way of preventing disease. I cannot help but think of the work of Dr. Miller, which has made him our highest scientific authority on the etiology of dental caries. His work has been solely directed toward the eradication of this destructive disease of the human teeth, and he is still working at it. All of his efforts are now being directed to the discovery of some agent which will prevent the destructive tendency of dental caries. It seems to me that what he has accomplished means something. His work is not out of date, it is still classic and standard and it will be a classic for many years to come. There are doubtless many other things to which the application of this knowledge will be brought in time, and we shall use it all for the purpose of benefiting humanity. We are making more progress than is apparent and there are many things to encourage us to go on. Dr. Smith says he does not mean to take a pessimistic attitude toward this matter, but it strikes us that he does. I am glad, however, to have him here with his strong personality and enthusiasm, which I hope may make such a deep impression upon our minds as to make us truly professional men rather than mere mechanics.

Dr. George Cook, Chicago: I have been wondering, since hearing one of Dr. Smith's statements, just what my teeth have done for me. They have certainly given me a great deal of pleasure, because of my grand and enormous size. I presume it is not what I have chewed with them that has produced this result, but it is the pleasure I have gotten out of them.

The essayist certainly touched up some very important points and has taken some views that perhaps many of us have lost sight of, and on the whole he may do us a great deal of good by giving us this pessimistic paper optimistically.

The essayist states that in 1839 we had one college, but now we have a great number, intimating that the number was now too great, because in '39 we only had one and got along with it, but he forgets that in '39 we did not have so many folks. The population of the Unitde States is now almost two-thirds more than it was in 1839. I don't think that we need be discouraged because of this fact anyhow. We are coming to treat our patients prophylactically, and this will take lots of time and require more men to do it, because it takes a lot of my times 'to clean a tooth well. I can fill two or three teeth usually while I am cleaning only one real pretty.

A criticism is offered on the literature and text books for teaching. I fully appreciate the many facts Dr. Smith has mentioned are true. As Dr. Hoff has said: The literature has not been, possibly, what it ought to be, but from the reading of Dr. Smith's paper as he commented on the saliva, I see he has evidently drawn pretty freely from that literature.

I feel that Dr. Smith's treatment, so-called prophylaxis, is really treating disease. I do not think we should call it prophylaxis treatment. When I was a small country lad and lived in the country with a doctor, I was taken once as assistant in a surgical case, and had to hold a lamp, made of grease with a lamp wick, while the physician made Cæsarian section for a woman in a farmhouse, with none of our present day antiseptic environments. The mother and child both lived, I have often wondered why, with so little knowledge of antiseptic principles or practice.

We have got to take care of our bodies that we may protect the teeth to some extent. All diseases of the mouth are almost purely local, but we have no diseases unless there is a predisposition existing in the individual before that disease is contracted, so that we have to go further back than simply scraping off the tartar and taking a pine stick and swab around the tooth for a while, saying that we are prophylactically preventing decay, pyorrhea and everything else. We are not, because when we have cleansed it thoroughly the mucus will get back and soon houses a great multitude of bacteria, The progress of dentistry has revealed this condition to us and has made it possible for Dr. Smith to found this great school of prophylaxis.

Many members of our profession who have not contributed anything in the way of sacrifice have given the most comforting relief from suffering that the world has ever known. Dr. Miller and Dr. Black and others have made great sacrifices as we all know, for the benefit of science of dentistry. There are plenty of men in the professon who are making almost heroic seacrifices every day; and I wondered when Dr. Smith was referring to the case of the little child on the street car why he did not get off the car and take the child to the infirmary where they would take care of its teeth and rescue it from its mother and the calamity.

Usually the sacrifices that I make professionally and otherwise give me a compensating pleasure. I do not come to these meetings because there is a great revenue in it, but to get something that I can take back home and utilize in the practice of an art that has done something for humanity in the past.

I do not wish to discredit the special emphasis of Dr. Smith's paper, but his tone and attitude seems to me rather discouraging to a young man who supposes that he is entering a useful field of work. Those of us who have labored so conscientious'y for so many years, like Dr. Smith and myself, are not worrying so much about our personal feelings in this matter, but we would like to leave the profession encouraged enough to go on and finish up this job of saving people's teeth. I would advise you not to get discouraged, I mean all you fellows back there in the room, because this prophylaxis treatment is not going to cure all the decay of the teeth; you will not lay aside your pluggers for a long time, neither will you do away with your artificial denture for a long time to come, much as I hope it may come. I tell you, gentlemen, there is a great deal involved in plugging a hole in a tooth. The greatest skill that human hands has acquired has been produced by the hands of dentists. No class of men has come into the world that can do better and more varied technical work than the dentist. No fingers have ever been trained, outside, probably of a few jewelers, that can do even the technical things that have been accomplished by dentists. I say that with all regard to the man who preaches prophylaxis, that it should be one of the essential parts of our practice, but, prophylaxis in its truest sense, has got to begin before the disease has fully established itself, and then it is prophylaxis.

I believe the majority of us has got the wrong idea of this prophylaxis treatment. Dr. Smith tells us that saliva is no more than water. I do not know where he gets his authority for this statement, but I presume he thinks that it is true, but he is mistaken. There are organic and chemical constituents in saliva that are important elements of this prophylaxis treatment. It is our environment, our conditions of life, that makes us predisposed or immune to disease, regardless of whether it is the enamel of the teeth, or whether it is the epithelial structure of the mucous membrane. I believe that true prophylaxis goes further back, goes back, even to the very origin of life itself; because we find that all animals naturally are immune and all plant life is naturally immune to disease, and by certain environments they become susceptible and disease is contracted. I do not believe that we are always going to prevent pyorrhea or caries of the teeth by prophylact'c means. I do not want to discourage your prophylaxtis treatment, or your efforts at keeping the mouth clean, but you can produce erosion of the teeth by simply using the tooth brush and rubbing the teeth with sticks. I can produce degeneration of the epithelial cell in six weeks in a dog's mouth with a tooth brush. Scaling the teeth occasionaly is an essential thing in a great many cases, but if we have the body as a whole in a perfect functional condition, I do not believe that we need to fear so much about our teeth if we keep them reasonably clean.

I was talking with an old fel ow that lives in my neighbor hood who is 84 years old, and I said: "How many teeth have you got, uncle?" "Every damn one," said he. "Hain't lost one yet." I said: "Ever used tooth brush, powders and mouth washes?" "Not a damn bit," says he, "I take a l'ttle whisky in my mouth once in a while, but that is the only thing that has ever passed my mouth, 'ceptin' somethin' to eat."

While I do not agree with all that Dr. Smith has said, yet I am glad he has said it.

There is one other point that I want to touch on, that is the point on dental literature. He made the statement that the sentiment of all our literature was largely shaped by the dictum of the trade journals. The *Dental Cosmos* probably has as much influence in the production of current dental literature as any publication in the country. Now I do not agree altogether with the fact that we are in the hands of the enemy, or the dental supply house, because Mr. White, who is dead, founded a dental journal as well as a great supply house and gave it the profession. I do not feel that we can lay all the literature that is bad to that particular cause. Dr. Hoff mentioned the fact that we were not in a literal sense wholly cultured, and yet that we d d have some culture. I was glad that Dr. Hoff held us up some. I have always been associated with physicians, my first conceptions of the healing art was that of the physician, then I knew nothing of dentistry, except that when the Doctor would be away, I would extract teeth for such colored people as would let me, as an amusement. I have a broader conception of medicine now, as I have had to spend years in hospital work, and in bacteriological, histological and chemical laboratories with medical men, so that I know something of what they do and what they have done. And I know further that dentists, Dr. Miller, more than anyone else, have made the nearest approach to the scientific knowledge of, at least, one pathological condition, that of caries of the teeth, for which there are few paralells in medical knowledge. We have many points that are not cleared up, as Dr. Hoff has said; probably they may not be very soon. I don't think we have any cause to be discouraged, as to what the dental profession can give us in the way of culture and study. The literature that is published in the dental journals, while it is not always pure or free from taint, nevertheless gives some evidence of literary skill if it does show up some faults on the part of the profession. In the great number of publications that have been brought out we have got together a good deal of valuable literature, and probably in some papers that Dr. Smith has quoted, if he had quoted some other paragraphs in them he would have shown that there was some real meat to be had. But I think he looks at the thing with a critical eye rather than from a broader search after true knowledge. This is the way it looks to me; and I want to say, in behalf of the *Dental Cosmos*, and it is nothing whatever to me personally, but it has published some of the most valuable literature the profession has produced, possibly much of it has been mechan ical or technical and it may be that every thought has been evolved or started in mechanics. There is nothing in the progress of human existence that has not passed through the mechanical stage somewhere, and we probably are swaying a little further toward mechanics in our profession than we ought, and I can agree very largely with some of the statements the essayist has made in that respect. People come to us with a tooth decayed and pulp exposed and in pain, and we at once proceed to put that person in a comfortable condition. We have got to fill teeth; that is what the public demands of us, as that is what the public needs most. As soon as you can get the teeth quiet, then with your little hoes and scrappers and rags and things you can go to work and polish them, so that they can afterward keep them clean.

As I said before, I appreciate this paper very much, as it has brough out a great many excellent po'nts, and I take issue with it purely, in a friendly discussion, and not in a slurring way. I respect and honor Dr. Smith for his efforts and sacrifices, but I thought I ought to say something against it because I did not just like the tone, not that I objected so much to what he said, as the way in which he said it, and the emphasis he put on some of its express'ons.

Dr. W. H. Jackson, Ann Arbor: If you were to go to any school and apply for a position, if you claim great scholarship in half a dozen different subjects, and you claim that you are qualified to teach any one of them, they will not want you at all as a teacher of any subject. Now Dr. Smith has taken up one subject and is fit to teach that subject and he does it well.

Take the people as they come to us. They have diseased and decayed teeth, and in order to correct these conditions we have to have the utmost skill in manipulation in order to treat them successfully. It requires just as mush intelligence and just as much knowledge to treat that class of cases as it does to treat the class of cases to which Dr. Smith has referred. We have a peculiar disease, and that disease has to be treated in a peculiar manner.

Another point I would like to call attention to is this: All pyorrhea trouble is dependant on a condition of the system which makes the tissues weak and causes the gums to break down. You will almost always find this trouble associated with a rheumatic or gouty diathesis. If your case arises from a constitutional condition, local treatment may help it, but it will not cure your disease. The systemic disease has to be cured in order to cure the local condition, and you have to treat constitutionally. You have to have a prophylaxis of the system, as you might say, before you will cure that disease. If the case arises from local causes, then your treatment by prophylaxis will cure the disease; if not, it is a constitutional disease, then no kind of local treatment will cure that disease, it will keep on recurring.

DR. E. B. SPAULDING, of Detroit: Dr. Zederbaum said there was a squad of prophylactics in Detroit. I happen to belong to that squad, and I presume many of my associates will recall my speaking Dr. Smith's name a good many times. I do not feel that I am capable of criticising Dr. Smith's paper because of my experience with his other papers. The first time that I read one of his papers I thought it was the queerest jumble that I had ever read, and I did not get much out of it, but I laid it aside, and read it again and again several times. I think about the tenth time that I read that paper I got a great deal out of it, and I was very pleased to find out later that Dr. Smith said it took him two years to write that paper. This paper that he has read to us to-night is like it, and will bear something more than a casual reading or hearing. I am sorry that it is not the privilege of every member here to see what a few of us saw in Dr. Smith's office some two or three months ago. He gave a demonstration that I do not think can be duplicated by any man in the profession of dentistry. From ten o'clock in the morning until about half past four or five in the afternoon, (he did not allow us to go to lunch), he had patients coming in at least every fifteen minutes, to demonstrate what he had been doing for the past five or six years by his method of prophylaxis treatment. He not only showed us mouths that were most beautifully healthy, but reclaimed from conditions which it was very apparent had been the subjects of pyorrhea in extreme stages; he also exhibited mouths all perfectly healthy that he was maintaining in that condition, and showed us that he was not only a polisher of teeth, but that he made gold fillings the like of which I have never seen before. I think all of those who saw them will bear me out in that statement. They were most beautiful, and any work he does is done in the most careful and perfect manner. His bridge work was unique and different from anything that most of us had seen before.

The man who made the statement that Dr. Smith was the best all around dentist he ever knew, ought to know what he was saying, because he worked with Dr. Smith for several years in his office. I only wish that Dr. Cook and all of you could have seen what we saw in that exhibit of results, you would certainly see that it meant something more than "a stick and a rag" treatment. Dr. Smith has high conceptions and ideals and he comes nearer realizing them than the most of us.

Dr. D. D. Smith: I have but few words in closing; let me first try to correct the impression that seems to have been received that I look upon this subject from a pessimistic standpoint. I said in the beginning that I am not a pessimist, on the contrary, I am extremely optimistic. I told you that I believe dentistry will one day stand as the right bower of medicine. I believe that is coming to pass. In what I have written, I desire simply to focus your attention upon some important matters which the profession in its self glorification has overlooked. I know that when a man is invited to read a paper on any dental subject he is rather expected to tell his auditors that we belong to the medical profession, are indeed a branch of it, that we have made the most wonderful improvements and advances in the profession possible. In proof of this he may point to our engines, to our pluggers, our furnaces for porcelain work. "See," he says, "what wonderful advances we have made," the essayist generally comes in that spirit. Now, gentlemen, it will not do for us to look always at things from that standpoint, for you and I will go into our offices from this time and we will find that in our little home circles we do not occupy altogether such exalted positions as we fain would believe. We might, when we get together, better consider the position of dentistry from a rational standpoint.

Perhaps the view which I presented may seem gloomy to you, but I did not mean to have it so. If we rightly consider these things, this view will do us good. The presentation of this paper was for the *help* of young men, not to discourage. Dr. Cook, in his good natured, facetious, rambling, has intimated that we cannot prevent mouth diseases, especially this one disease, caries, which dentistry is forever fighting. I tell you and Dr. Cook, however, after an experience of eleven years in preventive treatment, I am convinced that we can prevent it. Do you ask me if we can prevent all decay? I answer, No. But we can stop 90 percent of it, and if that is so, is it not worth while? It certainly is not done by any other means than by the prophylaxis treatment. This treatment properly administered will do one thing more, something that none of the old methods have ever been able to do, it will prevent the occurence of alveolar pyorrhea; it will cure it where it is established and absolutely arrest all recurrence. Alveolar pyorrhea is not properly a disease, even though my good friend Dr. C. M. Wright, of Cincinnati, says he does not agree with me in that. Dr. Wright says, "I love to talk about prophylaxis, to read about it, to dream about it—but to practice it as it should be practiced, Well! like Bill Nye, 'nothing pleases me more than to sit on a rail fence in the shade of a tree and watch my fellow man engaged in manual labor. Its inspiring.' "

If Dr. Wright would come down off the fence, hang up his coat and get honestly at *work*, he would soon learn the meaning and benefits of the "oral prophylaxis treatment." He would soon find, that although alveolar pyorrhea is uninfluenced by constitutional medication, diet, or talk, it is influenced and cured by what he loves "to talk about, to read about and to dream about," namely, the "prophylaxis treatment," practiced as it should be practiced.

Notwithstanding, we have Dr. Wright on the fence in the shade criticising. Alveolar pyorrhea is better defined as an inflammation and this inflammation we can and do arrest and prevent absolutely by our methods of local treatment. I am sorry Dr. Cook betrays such lack of familiarity with scientific dental practice. The "stick and rag" may do for the big feet of Chicago, but that practice is not in vogue in Philadelphia, and I am persuaded it is not here in Michigan. In attestation of this let me call to your attention the beautiful and most successful results that were exhibited this morning in your clinic by Dr. Rogers and Dr. Wood. Dr. Rogers presented, as you know, a unique and convincing demonstration of the power of the prophylaxis treatment to cure pyorrhea under most adverse conditions. All honor to Dr. Rogers. Dr. Wood's clinic was equally successful and convincing. They are both deserving of the highest credit for these most instructive exhibits. In the interest of general practice allow me here to correct the false impression that my practice is given wholly to the prophylaxis treatment. Possibly I turn away more patients every year than would make most young men a good practice, nevertheless, I do everthing in the line of dental practice that comes to my office. I have given myself and my energies for the past ten years largely to this work because I know it holds vast benefits for humanity. I want to see the dental profession moving forward; I want to see the young men in our profession elevated to a position where they shall command the respect of the medical profession. I respect the man who is able to accomplish the best results in the making of plates and in the use of gold, amalgam or porcelain in filling teeth, or who is able to do the best in any other department of dentistry. I am not decrying the mechanics of dentistry at all, and I am sorry if you have received the impression that I do. I believe in and exalt this department of dentistry which I lay before you, because it means so much more for the profession and for humanity. It ought to be known and accepted by every practitioner. It is practical, it is feasible, but it will require steady patience and tact. Know this, if you are intending to throw your energies into it and try to bring every patient under the influence of it as I have tried to do, that it took me two years after I was thoroughly imbued with the spirit of it to get twenty-five patients regularly under the treatment. Thus you can see some of the difficulties I have had to encounter; but I have at last succeeded in making my office a place of consultation rather than a dental workshop for receiving and executing orders. I have been misunderstood, misrepresented and maligned both in the profession and out of it, but truth has gloriously triumphed, the day is breaking, the way has been paved for you to higher achievements, and if you will but accept it, to a truly useful and successful career.

With this explanation I hope I have given you a better understanding of my position, if I have seemed too pesimistic, as Dr. Cook said, it is not exactly what I said, but the way in which I said it, you must remember that I am of a bilious temperament and I may see thing from a "bilious" standpoint when I do not mean to. If I have severely criticised dental literature it is because it deserves it. I have labored to be just, as I am optimistic in my views of dentistry. The time will come when gentlemen within the sound of my voice (I shall probably not live to witness it) will see among medical men a better comprehension of the pathological states and conditions of the human mouth and teeth in their bearings upon general health and disease, and then full recognition for the important and indispensable service of an awakened and enlightened dentistry will be made.

